# Interdisciplinary planning as a landmark for treatment: Case report with a 2-years follow-up

**DOI:** 10.1590/2177-6709.23.6.41.e1-12.onl

**Published:** 2018

**Authors:** Roberto Perasso, Monica Imelio, Renato Alcidi

**Affiliations:** 1 Private practice (Novi Ligure/AL, Italy).; 2 Private practice (Tortona/AL, Italy).; 3 Private practice (Alessandria/AL, Italy).

**Keywords:** Conoid upper lateral, Deep bite, Alexander discipline, Feldspathic ceramic veneer, Interdisciplinary treatment.

## Abstract

**Case report:**

young adult woman with esthetic complaints regarding her smile and frontal teeth aspect. At first glance, the problem seemed to be only the shape of the lateral upper incisors and a small diastema between the central incisors. The diagnosis shared between the orthodontist and the prosthetist led us to consider some other important aspects, such as the deep bite, the teeth inclination and the lips support. All these findings led us to consider that the right way to improve the esthetics of the patient’s smile was to plan an orthodontic treatment. This would serve not only for the distribution of the spaces, but mostly it would improve all other problems, before the restoration of the upper lateral teeth with two ceramic veneers.

**Results:**

the treatment plan achieved the right distribution of spaces for upper lateral incisors, significant correction of the incisors inclination with important reduction of overbite and better lip support, upper laterals restorations with ceramic feldspathic veneers, obtaining a good integration with natural teeth satisfying patient complaint.

**Conclusion:**

in cases which involve interdisciplinary approach, the fundamental step comes from the beginning, when only an initial diagnosis shared among the team of specialists can define the patient problems from different points of view. In this way, we can better understand the competency fields and plan the right treatment and time sequence.

## Resumo

**Relato de caso:**

paciente adulta jovem, sexo feminino, buscou tratamento devido à queixa com a estética do sorriso e o aspecto dos dentes anteriores. À primeira vista, o problema parecia ser apenas o formato dos incisivos laterais superiores e um pequeno diastema entre os incisivos centrais. O diagnóstico conjunto do ortodontista e do protesista fez com que considerássemos outros aspectos importantes, tais como mordida profunda, inclinação dentária e suporte labial. A partir de todas essas constatações, consideramos que a maneira mais adequada de melhorar a estética do sorriso seria planejar o tratamento ortodôntico. Assim, o tratamento serviria não apenas para melhor distribuição dos espaços, mas também melhoraria todos os outros problemas antes da restauração dos dentes laterais superiores ser realizada com duas facetas de cerâmica.

**Resultados:**

o tratamento planejado levou à correta distribuição dos espaços nos incisivos laterais superiores, com correção significativa da inclinação dos incisivos, importante redução da sobremordida e um melhor suporte labial, com restauração dos laterais superiores usando facetas de cerâmica feldspática, obtendo-se uma integração ideal com os dentes naturais, resolvendo as reclamações da paciente.

**Conclusão:**

em casos envolvendo abordagem multidisciplinar, as etapas iniciais são fundamentais, e apenas um diagnóstico inicial discutido entre a equipe de especialistas pode determinar os problemas do paciente sob diferentes perspectivas. Dessa forma, podemos compreender melhor os campos de competência e planejar a sequência e o tempo de tratamento mais adequados.

## INTRODUCTION

A 27-year-old woman came to our office to evaluate the esthetics of her anterior frontal teeth area.

Her complaint was her smile, the space between the upper central incisors, and the altered shape of the upper lateral teeth (conoid).

**Figure 1:**
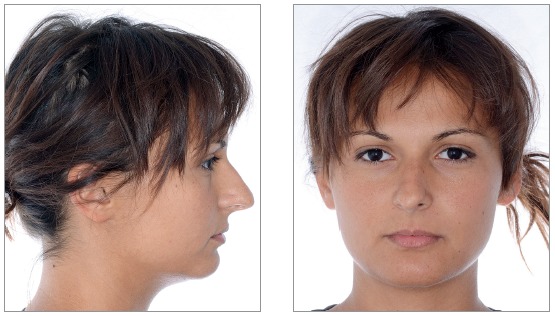
Initial facial views.

**Figure 2:**
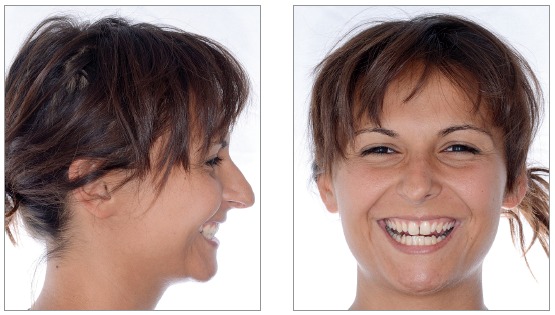
Initial facial views smiling.

**Figure 3:**
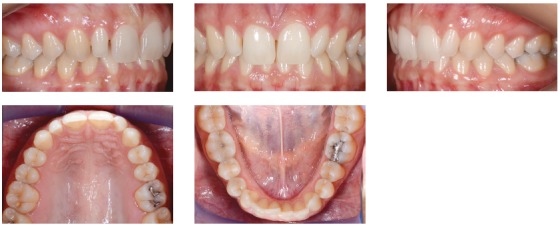
Initial intraoral views.

**Figure 4:**
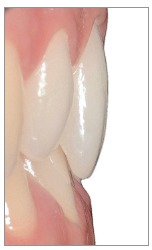
Initial intraoral views.

**Figure 5:**
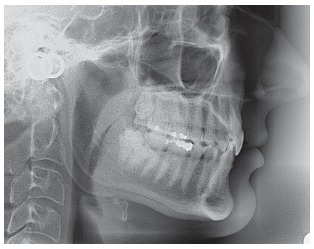
Initial lateral cephalometric radiograph.

**Figure 6:**
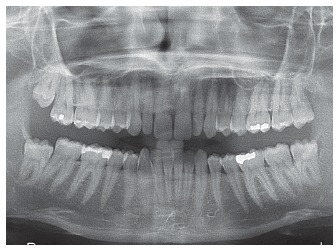
initial panoramic radiograph.

We identified the need for an interdisciplinary approach due to alterations of shape and teeth position. Photos, impressions and radiographs were taken at the first appointment (Figs 1 to 6).

## METHODS

### Diagnosis and etiology 

The presence of peg-shaped maxillary lateral incisors has been studied as a congenital malformation.^1,2,3^ The prevalence of this malformation has been found in 1.69% of boys and in 1.75% of girls; and it was associated with other dental anomalies, as follows: congenitally missing teeth, 31.8%; dens invaginatus, 19.7%; palatally displaced canines, 12.1%; supernumerary teeth, 7.6%; and transposition, 7.6%.^3^

In this case report we didn’t find any other dental anomaly associated with the two conoid upper lateral incisors.

**Figure 7:**
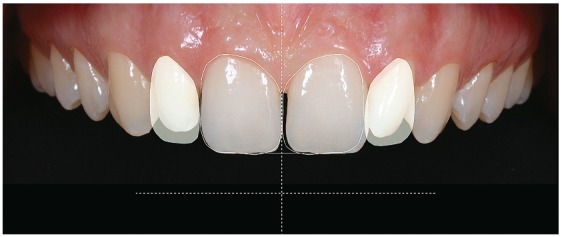
Initial DSD.

We made an initial DSD analysis for the interdisciplinary discussion (Fig 7).

Taking into consideration the esthetics in the upper frontal area, we considered that the space distribution and the position of the teeth did not allow us to harmonize the upper lateral incisors with the right shape and improve the lip support. These were the findings regarding the overall diagnosis:

Dental and skeletal Class I (upper limit, ANB = 4°), deep bite with severe short face (SN.GoGn = 22°, FMA = 15°, Y-axis = 65°), vertical position of the upper incisors (1.NA = 2°, 1-NA = 1 mm) with very steep anterior guidance, moderate crowding of the lower incisors (Figs 3, 4 and 5).

### Treatment objectives

Orthodontic treatment objectives were to open the bite, increase the vertical dimension^4^, reduce the overbite of the frontal teeth^5^, solve the lower anterior crowding and create the right symmetrical space for the reconstruction of the upper laterals. 

The issues for these restorations in an adult patient are the minimally invasive dentistry, a high biomechanical resistance and a long term stability. For this reason, we opted for ceramic veneers.^6^ ^,^ ^7^

In order to obtain a good light transmission, we decided to use feldspathic ceramic veneers.^8^ ^,^ ^9^ ^,^ ^10^

### Treatment alternatives

The approach to restore the frontal teeth without orthodontic treatment would have implied to do a more invasive prosthetic rehabilitation, in order to manage the spaces of the upper incisors, and it wouldn’t have corrected the deep bite.

Composites are the first choice for restoration in young patients due to its adaptability (to be modified with growth). However in this case of an adult patient, the composite wasn’t chosen due to its lower performances on the long term stability, in comparison with ceramic.^6^ ^,^ ^7^

### Treatment progress

1) Orthodontic treatment.
2) Bleaching treatment.
3) DSD to analyze and decide the desired lateral shape together with the patient.
4) Reconstruction of the upper laterals with two ceramic veneers.

#### 1) Orthodontic treatment

The Alexander discipline, a straight-wire technique, was used.^11^

The most important goal in the treatment was to correct the open bite by levelling the lower Spee curve. According to the Alexander discipline, this goal can be obtained by using an anterior bite stop.

**Figure 8:**
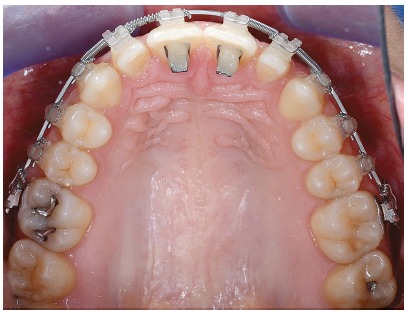
Orthodontic progress, occlusal view with bite blocks.

This could be a removable bite plane or, as in this case, two Bite-Turbo^®^ (Ormco™) applied on the palatal side of the upper incisors, followed by an application of some composite to increase the volume for a right contact with the lower incisors^11^ (Fig 8).

This anterior bite plane, by which we gain the space for lower bonding, and the rectangular lower arch (flat or with a reverse curve of Spee) together with the AD brackets prescriptions^5^ allowed us to level the originally deep curve of Spee^12^.

Another goal was to reduce the retroinclination of the upper and lower incisors (Fig 4-5) in order to obtain more space in the arches for the upper lateral incisorss and for the lower crowding solution^13^ ^,^ ^14^ ^,^ ^15^. This increase of the anterior torque would give a better support to the lips.^16^ ^,^ ^17^.

We evaluated that these goals would be well accepted from a functional point of view related to the reduction of the overbite and the steepness of the anterior guide, still respecting the functional area of the patient.^18^

The orthodontic treatment lasted 11 months and an immediate retention with fixed splint from canine to canine was applied, both on the upper and on the lower arches.

#### 2) Bleaching protocol

The patient asked for a brighter teeth color, therefore we performed a bleaching treatment in two steps before the dental restoration. The first step was a chairside starter application with 40% hydrogen peroxide gel (Opalescence Boost). The second step was a take-home whitening protocol with 10% carbamide peroxide (Opalescence PF) for two weeks.^19^

#### 3) Digital Smile Design

The DSD (Fig 14) can be helpful to find an ideal esthetic and drive the diagnostic wax-up^20^ (Fig 15). For a better communication with the patient, through the mock-up, we have created the shape of the wax-up directly in the mouth of the patient. We used a light-cured resin (Visco LC Anaxadent) and a transparent silicon (Anaxadent) positioned on a standard transparent impression tray. This way the light could pass through to cure the resin^21^ (Fig 17).

**Figure 9:**
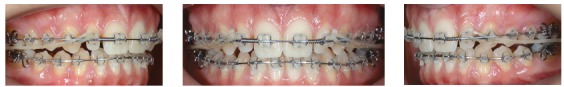
Orthodontic progress.

**Figure 10:**
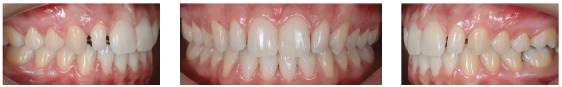
Final intraoral views.

**Figure 11:**
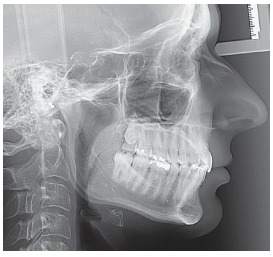
Final lateral cephalometric radiograph.

**Figure 12:**
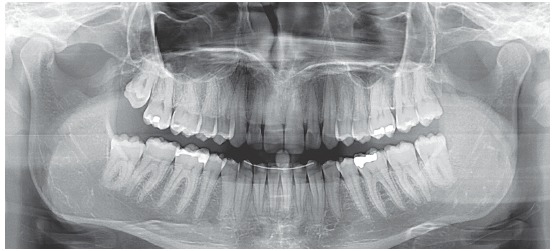
Final panoramic radiograph.

**Figure 13:**
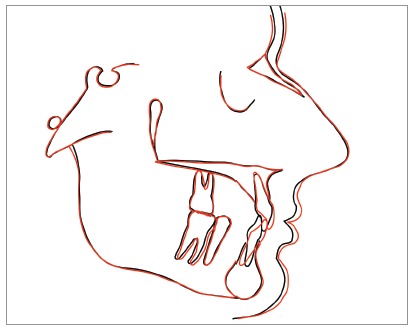
Total superimpositions of initial (black) and final (red) cephalometric tracings.

**Figure 14:**
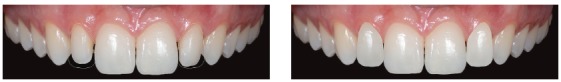
Final DSD.

**Figure 15:**
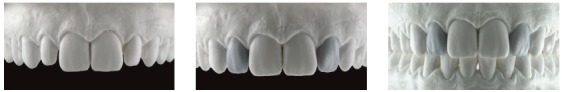
Wax-up.

**Figure 16:**
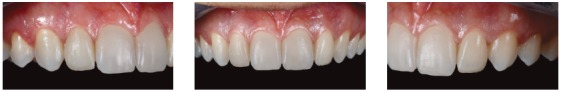
Intraoral mock-up views.

**Figure 17:**
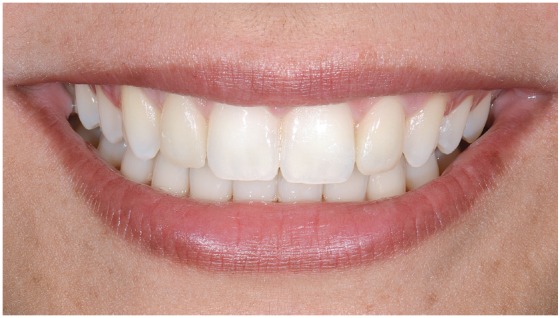
Smile mock-up view.

A new picture set (Figs 16 and 17) with the mock-up was useful for the team to discuss and choose the final shape together with the patient.

In this specific case the lateral shape is driven (Fig 16) by the natural central incisors and the patients' desire.

#### 4) Ceramic veneers

*Clinical preparation*

Every phase was executed with the intra-operatory microscope.

**Figure 18:**
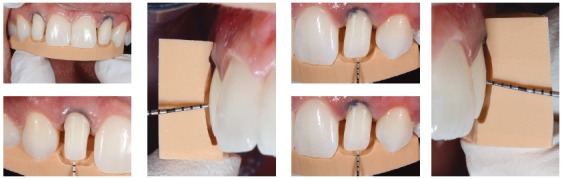
Clinical check of the spaces for veneers.

The silicon index was useful to perform a minimally invasive preparation (Fig 18), by which we removed an enamel thickness of 0,2-0,4 mm only in some cervical and buccal areas.

Every preparation edge was rounded, for the best fitting of the composite cement.^19^

Particular attention was observed for the undercuts in the interproximal areas.^22^

After a careful cleaning of the preparation areas, we took a one-step impression with polyether material (Permadyne Espe) on a customized impression tray.

The original transparent impression used for the mock-up was used also for the temporary restoration that was molded with acrylic resin (Coldpack A1). It was refined and fixed with this sequence: phosphoric (37,5%) etching for 30’’ in two or three small areas, cleaning with water for 60’’, enamel adhesive application and light-curing for 60’’ (Optibond FL).^9^

*Laboratory technique*

In order to perform an indirect prosthesis, we needed a working model with abutments, which accurately reproduced the position, surface and preparation margin, the adjacent teeth and the soft tissue around. The rising up profile of the restoration is important to condition and maintain the periodontal health.^23^

We used feldspathic ceramic (Creation CC Klema Meiningen) baked on refractory abutment (GC Orbit Vest Leuven, Belgium).

When an opaque layer to reconstruct tooth structure^9^ or to cover a dark abutment is not needed, the stratification begins directly with dentin color base mass.

We built up the teeth to the natural dimension obtaining the shape from a palatal silicon index of the wax-up.

**Figure 19:**
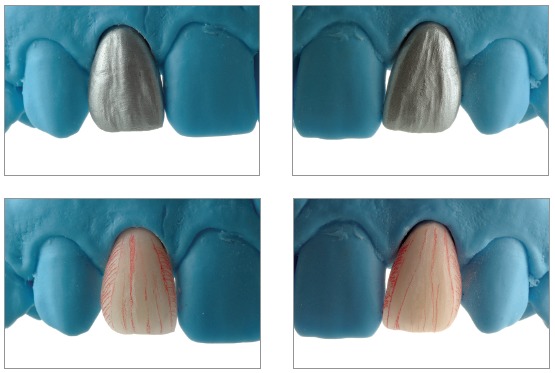
Surface texture of veneers.

The realization of the contour profile is simplified by the use of reference points marked with pencil (Fig 19), the enamel ridge and the transition angle lines are reduced or sharpened by using a diamond burr.

The vertical and horizontal surface texture is created with the help of surface colored powders (gold/silver) (Fig 19).

**Figure 20:**
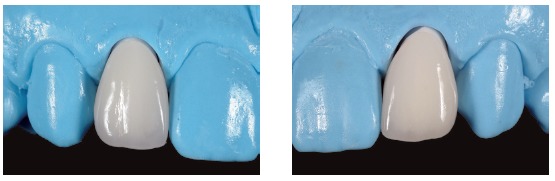
Surface finishing of veneers.

Once we had created a surface texture, we started the polishing phase by combining the use of both mechanic technique with diamond silicon polishers and glazing by oven. Then we mechanically finished polishing by using pumice powder or diamond paste, in order to obtain highly brilliant surfaces (Fig 20). 

*Veneers cementation*

The veneers cementation is a very important and difficult phase.

The preparations of the teeth proceeded one by one, with the following steps: cleaning with pumice and water, etching with 37% phosphoric acid for 20’’ (the adjacent teeth were protected with teflon tape), cleaning with water for 60’’.

After that, we applied primer and adhesive (IV generation, Optibond)) on the teeth and only adhesive on the veneers (already prepared with silane). The, we applied the light-curing composite (Enamel UD2), previously warmed at 55°C on the restorations.

Subsequently, we put the veneer on the tooth, with a slight and ongoing pressure, in order to remove all the excesses with brush, scaler, floss and plastic strip.

After the excesses were removed, we proceeded with the light-curing for 5 minutes from every direction.

Finally, we refined with straight blade chisel, finishing stripes, floss and rubber polishers, to obtain brilliant surfaces.^8^ ^,^ ^10^ ^,^ ^22^

The final retention, according to the Alexander discipline, was made by a wraparound retainer (its goal is to maintain the teeth position without occlusal interference) on the upper arch and by a fixed splint from 3 to 3 in the lower arch.^11^

Final photographs are presented in Figures 22 to 26, and follow-up photographs after 2 years can be seen in Figure 27.

**Figure 21:**
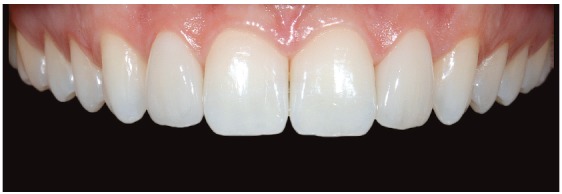
Close-up of final frontal teeth.

**Figure 22:**
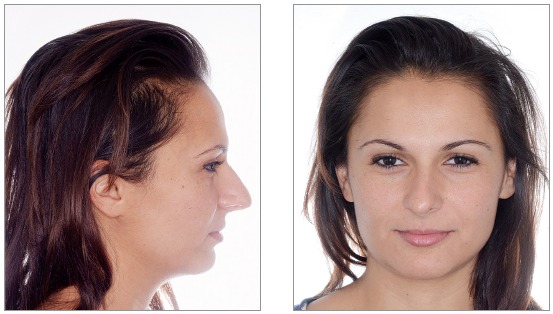
Final facial views with veneers.

**Figure 23:**
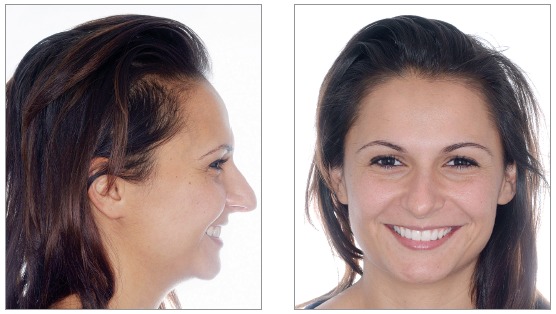
Final facial views with veneers.

**Figure 24:**
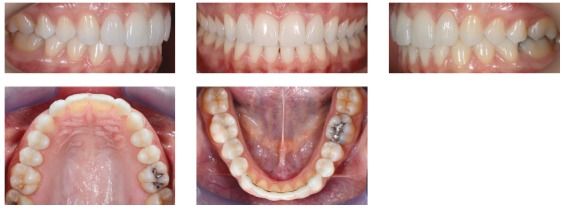
Final intraoral views with veneers.

**Figure 25:**
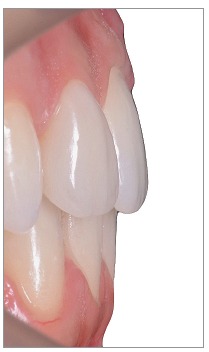
Final overbite and overjet.

**Figure 26:**
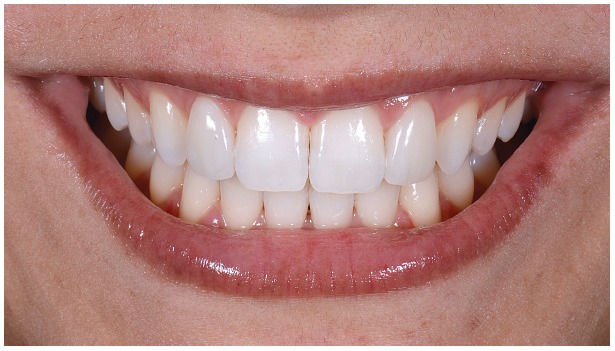
Smile with veneers.

**Figure 27:**
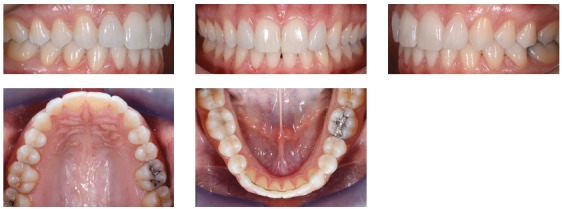
Two-years follow-up intraoral views.

## RESULTS

» Right and symmetrical distribution of the space for the upper lateral incisor (Fig 10).
» Alignment of the lower incisors
» No changes of the VD (Figs 11 to 13).
» Significant correction of the incisors inclination (1.NA from 2° to 11°, 1/1 from 157° to 137°) (Table 1), with important reduction of the overbite (Figs 11-26) and better lips support (Figs 11-13).
» Upper lateral restorations with good integration with natural teeth satisfying patient requests.

**Table 1:** Baseline (A) and final (B) cephalometric values.

Measurements		normal	A	B	A/B diff.
SNA	Steiner	82°	83°	83°	0
SNB	Steiner	80°	79°	80°	1
ANB	Steiner	2°	4°	3°	1
Wits	Jacobson	m = 0 ± 2 mm; f = 1 ± 2 mm	+1mm	+1mm	0
Angle of convexity	Downs	0°	+2°	+3°	1
Y-axis	Downs	59°	65°	65°	0
Facial angle	Downs	87°	88°	89°	1
SN-GoGn	Steiner	32°	22°	22°	0
FMA	Tweed	25°	15°	15°	0
IMPA	Tweed	90°	95°	103°	8
1.NA (degrees)	Steiner	22°	2°	11°	9
1-NA (mm)	Steiner	4mm	1mm	3mm	2
1.NB (degrees)	Steiner	25°	17°	21°	4
1-NB (mm)	Steiner	4mm	2mm	4mm	2
1/1 interincisal angle	Downs	130°	157°	137°	20
1-APo	Ricketts	1mm	-1mm	0,5mm	1,5
Upperlip-S line	Steiner	0mm	-4mm	-1mm	3
Lowerlip-S line	Steiner	0mm	-2mm	0mm	2

## CONCLUSION

A treatment plan which rises from an initial shared diagnosis is important in interdisciplinary cases. This allows the specialists to define the different points of view and better understand the competency fields, so as to plan the right sequence time for the treatment. 
